# Social determinants of health and patient outcomes in Long-term Acute Care Hospitals: A scoping review

**DOI:** 10.1371/journal.pone.0343885

**Published:** 2026-04-02

**Authors:** Christian M. Noval, Katherine Brooks, Leila Ledbetter, Erin Simon, Sharron Rushton, Donald E. Bailey Jr

**Affiliations:** 1 Duke University School of Nursing, Durham, North Carolina, United States of America; 2 Duke University Medical Center Library, Durham, North Carolina, United States of America; School of Public Health, University of Nevada, UNITED STATES OF AMERICA

## Abstract

**Introduction:**

Long-term acute care hospitals (LTACHs) are certified acute care facilities specifically designed to provide continuous specialized care and extended recovery for patients with chronic critical illness. LTACHs have high mortality rates because patients with complex care needs often experience fluctuations in their recovery trajectory; however, these outcomes are not solely driven by medically related factors. Social determinants of health (SDoH) contribute to up to 55% of all health outcomes; therefore, understanding how characteristics of SDoH influence patterns of health care utilization and health outcomes among patients in LTACH setting is crucial for optimizing outcomes in this population.

**Methods and materials:**

This scoping review utilized the U.S. Department of Health and Human Services *Healthy People 2030* SDoH framework to describe how SDoH influence outcomes for patients admitted to and discharged from LTACHs. The review covers the literature from 1982 to 2025 across four databases. The Joanna Briggs Institute (JBI) Manual for Evidence Synthesis guided the review and the PRISMA extension for scoping reviews guided the manuscript development.

**Results:**

Five studies met the inclusion criteria. Findings indicate that the current literature focused on SDoH within the LTACH setting has predominantly concentrated on only three of the five established domains: social and community context, economic context, and healthcare access and quality. Race was identified as a key demographic variable in three studies that examined its association with outcomes such as mortality, morbidity, readmission rates, and health care costs. LTACH location and insurance coverage represented health care access and quality, and income reflected the broader economic context.

**Conclusion:**

There is limited literature on this topic, and the review findings were insufficient to describe the complex interplay between SDoH variables and key patient outcomes. The degree to which the variables under each SDoH domain have been studied may not provide sufficient information to represent them. Comprehensive research on the effects of SDoH on outcomes of patients in the LTACH setting is warranted to explicate possibly complex causal relationships, inform targeted interventions to improve patient outcomes, and maximize the value of LTACHs, especially in light of the recent facility closures.

## Introduction

Long-term acute care hospitals (LTACHs), sometimes referred to as long-term care hospitals, are certified acute care facilities specifically designed to provide continuous specialized care and recovery for patients with chronic critical illness (CCI)[1, 2]. Patients with CCI have multiple chronic conditions and clinical complexities related to the acute phase of critical illness and require a high level of care that is responsive to fluctuations in their recovery trajectory [3–7]. According to the Medicare Payment Advisory Committee (MedPAC), in 2022, only 23% of Medicare beneficiaries treated in LTACHs were discharged to the community, 17% died during their LTACH stay, and 14% were deceased within 30 days after discharge [8]. A comprehensive analysis of 5-year survival rates among LTACH patients (N = 14,072) revealed that 48% of these patients were alive at the 1-year mark, and only 18% survived to the 5-year mark [9]. Such outcomes are not solely driven by medically related factors; nonmedical factors such as social determinants of health (SDoH) have been found to contribute to up to 55% of health outcomes [10]. However, it is not known how SDoH affect health outcomes and health care utilization patterns among patients in the LTACH setting.

SDoH describe the conditions in which people are born, grow, live, work, and age [10]. SDoH include individual-level determinants (e.g., educational level, employment status, housing status) as well as community-level determinants (e.g., environmental and neighborhood characteristics) that shape health as protective or risk factors [11, 12]. As SDoH are influenced by the overarching distribution of money, power, and resources, they contribute to health disparities and inequities that affect individuals’ health outcomes, functioning, and quality of life [13–15]. Among the various SDoH, socioeconomic factors such as poverty, education, and employment have the strongest impact on patient health outcomes [16]. Black, Hispanic, or American Indian and Alaska Native race is a factor that has been linked to disparities in life expectancy, health status, chronic diseases, and access to health care [17]. In critical care units where most LTACH patients originally received care, SDoH such as insurance coverage and race have been shown to moderate health outcomes and mortality [18, 19].

Most of the epidemiologic work conducted in LTACHs has described facility characteristics or clinical risk factors, patterns of utilization, or care outcomes of patients (primarily Medicare beneficiaries), who constitute two-thirds of LTACH patients, but not the effects of SDoH [6,9,20–26]. The *Healthy People 2030 Framework* emphasizes the importance of identifying and potentially mitigating health disparities in order to promote health equity and enhance the overall health and well-being of all individuals; within the framework, key SDoH domains include social and community context, economic context, access to education and its quality, neighborhood and built environment, and access to health care and its quality [14]. Given that patients in LTACHs have complex care needs and require extended recovery, it is imperative to examine how SDoH characteristics affect their patterns of health care utilization, health outcomes, and mortality. Therefore, this scoping review aimed to describe how and the extent to which the literature has explored outcomes of patients admitted to and subsequently discharged from LTACHs in relation to their SDoH.

## Review methodology

The overarching goal of this review was to examine how the SDoH of adult patients admitted to and discharged from LTACHs have been explored in the literature. Research questions were as follows: “What SDoH variables have been studied in patients discharged from LTACHs?” and “What relationships between SDoH and discharged patients’ outcomes have been identified in the literature on LTACHs?” A scoping review methodology was selected because the goal was to explore the existing literature and identify key determinants [27]. The Joanna Briggs Institute (JBI) Manual for Evidence Synthesis was utilized to guide the review [28]. The PRISMA extension for scoping reviews (PRISMA-ScR) was used to guide manuscript development [28, 29]. The scoping review’s protocol was registered and updated with the Open Science Framework (OSF) (https://doi.org/10.17605/OSF.IO/EZD7T).

### Eligibility

The Population, Concept, Context (PCC) framework [30] was utilized to formulate the research questions for this review. The population was adult patients, the context was LTACH, and the concepts were the SDoH domains and patient outcomes. Inclusion criteria were studies (1) of patients 18 years and older who were admitted to and discharged from a LTACH or long-term care hospital in the United States, (2) describing SDoH domains and patient outcomes, and (3) published in English (or an English translation). In the United States, LTACHs are post-acute, reimbursement-driven, free-standing, or co-located care settings focused on extended acute care [31]. This review excluded studies of LTACHs outside the United States because, in other countries (e.g., Canada, the United Kingdom, Japan, Australia), LTACHs may be integrated into specialized weaning units, rehabilitation, and community care services [32–35]. The review protocol was updated prior to title and abstract screening to focus on patients admitted to and subsequently discharged from LTACHs to align more fully with the goals of the review. Table 1 describes the study’s final and comprehensive inclusion and exclusion criteria.

**Table 1:** Scoping review inclusion and exclusion criteria.

	Inclusion	Exclusion
**Population**	• Adult, 18 years and older	• Younger than 18 years old
**Context**	• Patients admitted and discharged from LTACHs or LTCHs located in the US.	• Acute care (e.g., hospitals) • Lack of sub-analysis on LTACH variables in studies where other post-acute care setting was included (e.g., SNF, IRF, LTC, ALF).^b^
**Concept**	SDoH Domains ^a^ *• Social and community context* (e.g., ethnicity, race, religion, safety, gender discrimination) *• Economic context* (e.g., employment, income, poverty, food security) • *Education access and quality* (e.g., educational attainment, literacy, English proficiency) • *Neighborhood and built environment* (e.g., housing, transportation, food and water availability, environmental conditions, sufficiency of social services) • *Healthcare access and quality context* (e.g., access to healthcare, access to insurance,healthcare laws, attitude towards healthcare) Patient Outcomes **•** Discharge locations (e.g., home, home care, SNF, IRF, ALF, readmitted to hospital) • Survival, alive or dead **•** Length of stay in LTACH. • Functional abilities	• SDoH domain or variable is not the focus of the study nor one of the main objectives of the study.

^a^SDoH domains adapted from USDHS’ *Healthy People 2030 Framework* [14].

^b^Skilled Nursing Facility (SNF); Inpatient Rehabilitation Facility (IRF); Long-term Care (LTC); Assisted Living Facility (ALF).

### Information sources

The four databases searched included (1) MEDLINE via Ovid, (2) Web of Science via Clarivate, (3) Embase via Elsevier, and (4) ProQuest Dissertations and Theses to capture pertinent grey literature. Other sources of grey literature were the Agency for Healthcare Research and Quality [36], MedPAC [37], and the Centers for Medicare and Medicaid Services [1].

### Search strategy

In accordance with the Tax Equity and Fiscal Responsibility Act of 1982 (TEFRA), Congress established LTACHs as recipients of a prospective payment system with fixed and predetermined rates, similar to acute care hospitals [6, 38]; therefore, this scoping review on SDoH and patient outcomes in LTACH covers the literature from 1982 to 2025. The comprehensive search strategy was developed and conducted by professional medical librarians (LL and ES) in consultation with the authors and included keywords and subject headings representing LTACHs and patients. The searches were validated against a set of preselected articles and independently peer-reviewed by another librarian using a modified PRESS Checklist [39]. A manual search on AHRQ, MedPAC, and CMS portals did not yield any articles pertinent to the review. Table 2 provides a detailed and reproducible search strategy, including date ranges and search filters for Medline. The complete search strategies are presented in the supplementary material (S1 Appendix).

**Table 2:** Medline (Ovid) search strategy.

Set #	Search Strategy	Results 1/30/24	Results 2/10/25
1 LTACH	(exp “long-term care”/ or (“long-term care” or “long term acute care” or “long-term acute care” or “long-term care” or “long term care” or “longterm care” or “long term ventilation” or “long-term ventilation” or “longterm ventilation”).ti,ab.) adj3 (exp hospitals/ or (hospital or hospitals).ti,ab.)	4034	4,120
2	(LTACH or LTCH).ti,ab.	213	235
3	(“long term ventilation” or “long-term ventilation” or “longterm ventilation”).ti,ab adj3 (facility or facilities or unit or units).ti,ab.	4	5
4	1 or 2 or 3	4091	4,190
5 patients	exp patients/	85007	88,294
6 patients	(patient OR patients OR client OR clients OR clientele OR admitted OR admit OR admits or individual or individuals).ti,ab.	9719919	10,315,623
7	5 or 6	9555308	10,340,803
8 LTACH and patients	4 and 7	2629	2,699
9	(exp Canada/ OR exp Greenland/ OR exp Mexico/ OR exp Africa/ OR exp “Central America”/ OR exp “Caribbean Region”/ OR exp “Latin America”/ OR exp “South America”/ OR exp Asia/ OR exp Europe/ OR exp Oceania/)	3472259	3,624,688
10	8 not 9	1741	1,790
11	10 not (case reports OR editorial OR letter OR comment).pt.	1652	1,698
12 updates	limit 11 to yr = “2024–2025”		68

The initial search was conducted on January 30, 2024, with an additional search of ProQuest Dissertations & Theses Global on February 19, 2024. The searches for all databases were updated on February 10, 2025. Search hedges or database filters were used to remove publication types such as editorials, letters, case reports, and comments, as appropriate for each database. The reference lists of the final articles included were reviewed, and citation tracking using Citation Chaser was employed to identify 119 relevant studies, which were added to the title and abstract screening stage [40].

### Source of evidence selection

**Fig 1:**
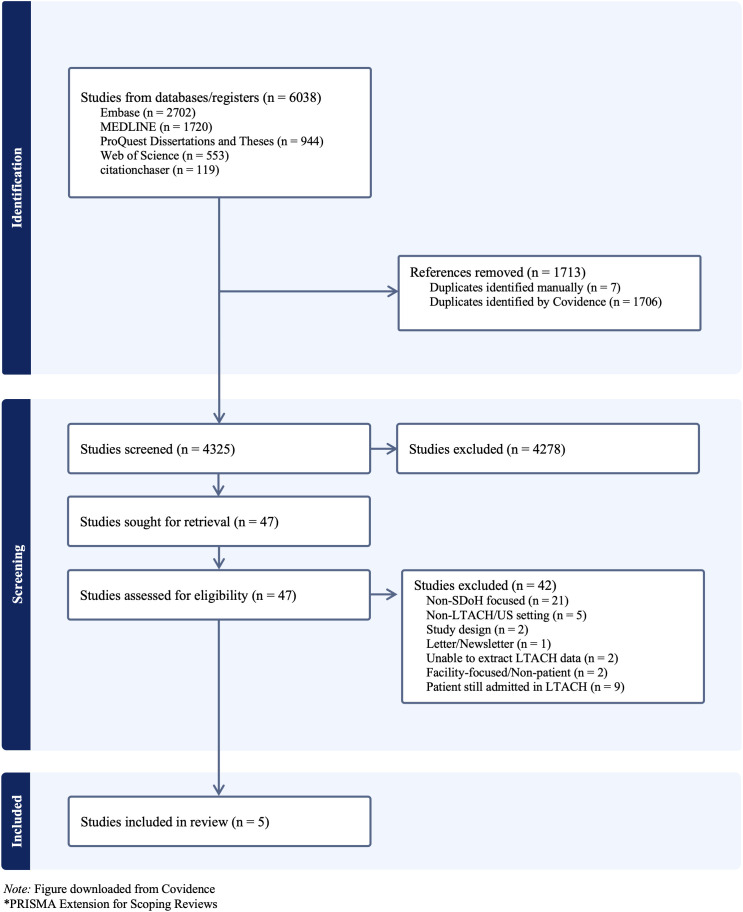
PRISMA-ScR* flow diagram for the study selection process.

The results of the comprehensive search were subsequently imported into Covidence, a web-based platform designed to manage systematic reviews and other literature reviews [41]. Through Covidence, duplicates were efficiently eliminated, resulting in a refined dataset with unique citations available for subsequent screening (Figure 1). Prior to title and abstract screening, a pilot screening was conducted with 50 randomly selected articles to ensure consistency in the source selection process. The study selection was carried out independently by two authors (CN and KB) against predetermined inclusion and exclusion criteria. Studies were excluded if they did not meet the inclusion criteria based on title and abstract review. Similarly, a pilot study was conducted in the full-text screening stage, and papers were reviewed in detail by two independent reviewers and excluded if they did not meet the inclusion criteria. All disagreements were discussed and resolved by adjudication with a third reviewer.

### Data extraction

Articles that met the eligibility criteria underwent data extraction using a tool developed by the research team to collect elements needed to answer the research questions. A pilot test of the data extraction tool was independently conducted by two team members (CN and KB) on three eligible articles. With this approach, the team members audited the data-charting process, familiarized themselves with the data extraction tool, noted any attributes that could be refined, and established consistency in data charting between the authors [30]. The pilot test resulted in no changes to the data extraction tool.

After completing the pilot test, the same team members independently recorded the following data from the eligible articles on the data abstraction tool: (1) author/s; (2) year of publication; (3) study aims/purpose; (4) study design and endpoints; (5) study population utilizing the SDoH variables; (6) study setting/location; (7) patient outcome variables such as discharge locations (e.g., home, SNF), survival (alive/dead), length of stay (LOS) with their corresponding statistical findings (e.g., means, p-values, coefficient variables); and (8) other key findings pertinent to the review question, such as cost of hospitalization and diagnosis groups.

### Data analysis and synthesis

The identified articles were analyzed and synthesized by collating, summarizing, and reporting the data obtained from the data extraction and mapping these to the *Healthy People 2030* SDoH domains and the patient outcome concept. These data were presented in tables, and statistical findings for the studies included in this review were obtained.

## Results

The search resulted in 6,038 citations imported to Covidence, of which 1,713 duplicates were identified and removed. Of the 4,325 citations screened, a total of 47 studies proceeded to full-text screening, with one article included from the most recent search update. However, 42 of these were ultimately excluded. Reasons for exclusion are detailed in Figure 1, which lists the studies assessed for eligibility, and only five studies met the inclusion criteria.

### Study characteristics

Five studies met the inclusion criteria [42–46]. All were conducted in the United States between 2003 and 2019 (Table 3) and involved a secondary analysis of patients’ electronic medical records and/or Medicare and Medicaid beneficiary claims. Among these five studies, three employed retrospective study designs [42–44]. Notably, two of the studies were a thesis and a dissertation, respectively, neither of which had been published in a peer-reviewed journal [43,46]. The clinical characteristics of the participants in the included studies were diverse, including individuals on mechanical ventilators, requiring tracheostomy management, or experiencing multiple organ failure. Of the five included studies, four focused on the LTACH setting only [42,44–46]; in contrast, one study included a comparative analysis of demographic variables such as race, with outcome measures across three distinct post-acute care settings: LTACH, skilled nursing facility (SNF) and inpatient rehabilitation (IR) [43]. Subanalysis pertaining to LTACH was included in the review findings.

**Table 3:** Study characteristics.

Author (year of publication)	Study Design	Data Source	Total Sample (N)	Patient’s Age^a^	Study Setting
Dematte D’Amico JE, Donnelly HK, Mutlu GM, et al. (2003) [42]	Retrospective study	Patients’ medical records	285	≥ 18 years old	4 freestanding LTACH^b^ facilities within Chicago
Frank JP. (2013) Frank [43]]	Retrospective cohort study	Medicare Provider Analysis and Review (MedPAR) File data	1933	≥ 65 years old	LTACH, SNF, and IRF^b^
Kahn JM, Barnato AE, Lave JR (2015) Kahn, Barnato [44]	Retrospective cohort study	Patient level from Medicare Provider Analysis and Review (MedPAR)	391,292	≥ 65 years old	289 LTACHs^b^ in continental US; Free-standing (n = 192) and co-located (n = 187)
Cole ES, Willis C, Rencher WC, et al. (2016) [45]	Descriptive analysis	Georgia Medicaid claims data	458	≥ 18 years old	5 LTACH^b^ facilities in Georgia
Gibson S. (2019) Gibson [46]	Cross-sectional design	Patients’ electronic medical records	428	≥ 18 years old	1 LTACH^b^ facility in South Georgia

^a^Based on the study’s inclusion criteria.

^b^LTACH – Long-Term Acute Care Hospitals; SNF – Skilled Nursing Facilities; IRF – Inpatient Rehabilitation Facilities.

### Identified social determinants of health (SDoH) domains

This review indicates that the current literature on SDoH has predominantly concentrated on only three of the five established domains: social and community context, economic context, and health care access and quality (Table 4). The domains of education access and quality, as well as the neighborhood and built environment, were notably absent from the studies cited in this review.

**Table 4:** Characteristics of studies with identified SDoH, study outcomes, and key findings.

Author (year of publication)	Total Sample (N)	SDoH Variable	SDoH Domain*	Study Outcomes	Key Findings
Dematte D’Amico JE, Donnelly HK, Mutlu GM, et al. (2003) [42]	285	Race	Social and community context	• Patient’s survival at 28th day after LTACH admission	• African American race was not a significant indicator of mortality. • Significant predictors were age categorized into three levels (<60 years, 60–74 years, and >74), OSF score,** and APACHE 3 score.**
Frank JP. (2013) [43]	1933	Race	Social and community context	• Cost of LTACH hospitalization • Mortality and discharge location	• Patients transferred to another facility for further treatment; three quarters were White female patients • LTACH patients transferred to another facility had more expensive cost of treatment.
Kahn JM, Barnato AE, Lave JR (2015) Kahn, Barnato [44]	391,292	LTACH location (co-located vs free standing)	Healthcare access and quality context	• Mortality • LOS • 180-day hospitalization-related costs	• Adjusted mortality was higher among patients transferred to co-located LTACs, both at 30, 90 and 180 days (p = 0.6, p = 0.04, p = 0.04 respectively). • Costs equal between the two types of LTACH location (p = 0.51); no difference in LOS (p = 0.11).
Cole ES, Willis C, Rencher WC, et al. (2016) [45]	458	Fee-for-service Georgia Medicaid members	Healthcare access and quality context	• LOS • Place of discharge • 30-day hospital readmissions, and per-patient costs	• LOS at LTACH was 36 days, average LOS ranged from 29–40 days (across the 5 sites). • Discharge settings: Another PAC (37.2%) and Community (34.3%).
Gibson S. (2019) [46]	428	Race and Income	Economic context, and Social and community context	• 30-day hospital readmission	• Only length of stay was significant (LOS < 30 days significantly lower odds of being readmitted)

*Note:* LOS – Length of Stay; LTACH – Long-Term Acute Care Hospitals; PAC – Post-acute Care.

*Based on the *Healthy People 2030 Framework.*

**OSF – Organ Systems Failure; APACHE – Acute Physiology and Chronic Health Evaluation.

#### Social and community context.

Three studies included a social or community context. Race was identified as a key demographic variable in three studies that examined its association with outcomes such as mortality, morbidity, readmission rates, and health care costs (Table 4) [42,43,46]. Race categories were White, Black/African American, and Hispanic, except for one study which aggregated Asian, Hispanic, North American Native, other, and unknown into the “Others” category [43]. In a study on LTACHs in Chicago (N = 300), the majority of patients were Black/African American (61%) [42], in contrast to the Medicare data source (N = 1933), which consisted of predominantly White patients (75%) [43]. Additionally, one study examined the implications of race within specific patient populations (e.g., African American individuals with chronic obstructive pulmonary disease) and their subsequent readmission risk factors.

#### Economic context.

Only one study examined the association between income and patient outcomes. This study found income was not independently associated with readmission risk among African Americans with pulmonary disease [46]. Additional factors investigated as potential predictors of readmission included race and marital status (categorized as married, divorced, single, or widowed).

#### Health care access and quality.

Health care access and quality data were included in two studies [44, 45]. The first characteristic of health care access was the type of LTACH (co-located or free-standing) [44]. Kahn et al. [44] defined co-location as patients discharged to either a hospital that operates within an acute care setting (i.e., short-stay hospital) or to LTACHs located within a 1-mile proximity to these short-stay hospitals. The second characteristic of access was the patient’s type of insurance [45]. The third characteristic comprised services and costs associated with patient care in the LTACH [45].

As noted by Kahn et al., patients transferred to a co-located LTACH were likely to have been admitted to an ICU and most likely to be transferred for mechanical ventilator weaning. In terms of health care insurance, more than half of the patients studied (52%, n = 239) were dual eligible for both Medicaid and Medicare benefits [45]. Specifically, the outcomes of Medicaid patients were described by the type and volume of services, cost of care, and outcomes related to LTACH care [45]. Quality of care was assessed by mortality, cost of care, and risk for readmission (Table 4) [43–45].

### Relationship between SDoH variables and patient outcomes

The extant literature reflects limited exploration of the relationship between SDoH variables and patient outcomes among patients discharged from a LTACH setting. Empirical evidence indicates mixed results on the relationship between race and various outcomes across different studies and contexts (Table 4). In studies examining patients on mechanical ventilation (N = 1,933), LTACH patients were predominantly White (75%), likely to be transferred to another facility for further treatment (74%), and discharged alive after a follow-up stay (81.5%) [43]. Although there were more deaths among White patients (77%), only one patient in the “Others” category was alive at discharge from a follow-on stay (N = 81). Comparative analysis indicated that racial demographics did not serve as significant predictors of mortality, even in the sample predominantly comprised of African American patients [43]. Further regression analysis revealed insignificant associations among African American patient age relative to 30-day readmission rates (p = 0.53), with LOS in LTACH emerging as the only statistically significant variable (p = 0.03) [46].

The patterns of health care utilization were significantly higher among LTACH patients.

Patients admitted to a co-located LTACH required longer hospitalization and had higher incidence of hospital readmission (RR = 0.91 [0.84,0.98 95% CI], p = 0.02) and increased 90-day and 180-day mortality (RR 1.06 [1.00,1.13 95% CI], RR 1.05 [1.00,1.11 95% CI], respectively, p = 0.04). The LOS for these patients typically ranged from 9 to 40 days, with associated costs ranging from $1,574 to $46,805 (LTACH vs. SNF p < 0.0001, LTACH vs. IRF p < 0.0001) [43]. LOS of fewer than 30 days was linked to a reduced risk of readmission; however, more than a quarter of Medicaid patients who were discharged to the community (N = 458) were readmitted to acute care hospitals within 30 days [45]. Additionally, nearly half of the African American patients (44.5%, N = 428) reported an annual income ranging from $15,000 to $30,000, and the association of their income with 30-day readmission risk revealed no significant association (p = 0.16) [46].

In summary, this review identified five studies that explored SDoH and their relationships to patient outcomes in the LTACH setting. In the social community context, three studies assessed the link between race and patient outcomes, yielding inconsistent results [42, 43, 46]. Dematte et al. [42] reported that White patients had higher rates of transfer for further care and survival compared to Black/African American, Hispanic, and other racial backgrounds. Conversely, Frank [43] and Gibson [46] found no significant racial differences in rates of mortality and readmission, and costs of care. Within the economic context, only one study examined income and found no significant association with risk for readmission [46]. Additionally, two studies explored healthcare access and quality [44, 45]: Kahn et al. [44] associated co-located LTACH admissions with increased mortality, while Cole et al. [45] linked the length of stay under 30 days with lower readmission rates.

## Discussion

Among the studies included in this review, we identified three SDoH domains and four SDoH variables to address our research questions. The SDoH domains with their corresponding variables included social and community context (race), economic context (income), and health care access and quality context (type of LTACH location and insurance). Race was the most commonly reported SDoH variable in three out of five studies. In the reviewed studies, race has been utilized as a predictor variable for patient outcomes, including health care utilization, cost, and survival. However, the reviewed studies had mixed results on the association between race and key outcomes, suggesting that racial demographics may not inherently influence survival rates in this context. Race should not be construed as a risk factor or predisposition to adverse outcomes in this setting. It is important, however, to recognize that race reflects the underlying elements of social disadvantage, including disparities in access to health care services, which are intricately linked to health determinants and overall health outcomes [47].

One study in this review, under the health care access and quality domain, examined the variability in patient outcomes, specifically mortality rates and LOS, across LTACH locations [44]. The findings revealed statistically significant differences in outcomes based on the type of LTACH location. Nevertheless, it is noteworthy that over 50% of patients in both types of LTACH locations died within 1 year. The elevated mortality rate within LTACHs in the reviewed studies raises important considerations regarding their effectiveness, particularly in the context of patients with CCI, thereby underscoring the challenges of interpreting the potential benefits of LTACHs in reducing mortality in this population [20, 48]. However, during the COVID-19 pandemic, LTACHs played a critical role in managing health care resources by serving as overflow facilities for patients requiring extended acute and intensive care. This strategic utilization contributed significantly to alleviating the burden on acute care hospital beds, thereby facilitating overall health care delivery during a time of unprecedented demand [49, 50].

In the study on Georgia Medicaid Fee-for-service, patients with dual eligibility (eligibility for both Medicare and Medicaid insurance) were identified; however, they were not the focus of the study [45]. The absence of an examination of the relationship between dual eligibility and patient outcomes in the study significantly limits the understanding of potential associations. Patients qualify for Medicare based on age (65 years and older) and/or certain health conditions, including end-stage renal disease diagnosis, Amyotrophic Lateral Sclerosis, and disability [51]. In contrast, Medicaid eligibility encompasses low-income families, including older adults, individuals with high medical needs, or individuals with disabilities [52]. Studies have shown that dual eligibility may exacerbate disparities in access to care [53]. Moreover, a 2020 report to Congress by the Office of the Assistant Secretary for Planning and Evaluation identified dual eligibility as one of the most crucial social determinants impacting patient outcomes [54], thus dual eligibility may help elucidate why more than a quarter of patients discharged from LTACHs in the reviewed study subsequently transitioned to another post-acute care facility for skilled care and experienced increased readmission rates.

The variability in themes identified across the five studies, and the extent to which the variables pertinent to each SDoH domain have been investigated, may indicate a lack of comprehensive information needed to adequately represent domains. For example, although race has been identified as a key variable within the social and community context domain, the disparity in health outcomes between races in the reviewed studies can also be attributed to patient demographics and clinical characteristics (e.g., increasing age, severity of illness, prolonged mechanical ventilation, cognitive impairment) and the demographic composition of patients with CCI [9, 20, 23, 24, 42, 22]. Health care access, represented by type of LTACH (co-location and stand-alone) and patient’s insurance coverage, has been explored more extensively in relation to health care utilization, survival, and readmission risks instead of the health disparities that may contribute to differences in outcomes among patients with various SDoH [19]. Furthermore, the review did not find a strong association between socioeconomic factors and patient outcomes; only one study examined income within the broader context of economic factors [46]. Hence, it is imperative to exercise utmost caution when interpreting these findings, as other socioeconomic measures, including employment status and educational attainment, may significantly influence patient outcomes [16, 55].

This review examined SDoH in the context of outcomes of patients admitted to and discharged from LTACHs. Based on the limited literature on this topic, the review findings were not sufficient to describe the complex interplay between SDoH variables and key patient outcomes. Although disparities in health care utilization and mortality rates were evident, the influence of SDoH on patient outcomes warrants further investigation to unravel possible complex causal relationships and inform targeted interventions to improve patient outcomes. This knowledge might help (a) health care providers seeking to implement strategies to support patients as they navigate their recovery trajectory and health care decision-making, and (b) policymakers considering legislation pertaining to programs that increase access to social services, housing, and employment opportunities. Addressing SDoH entails creating social, physical, and economic environments that foster good health, well-being, and optimal health outcomes for diverse patients [14, 15]. The integration of SDoH into patient care plans may enhance the value of LTACHs, which is of importance given the multiple closures of LTACHs in recent years [56].

This review has some limitations related to both the methodology and the included literature. The first was related to the decision to use US-based studies. We decided to exclude non-US studies owing to fundamental differences in healthcare system structures and definitions of LTACHs, this decision inherently restricts the generalizability of our findings. Second, the search strategy was limited to four databases, which may have resulted in the omission of other studies pertinent to the research questions. Third, this review excluded long-term ventilator units and designated floors within acute care hospitals, as they do not fall under the classification of LTACH as defined by CMS. Fourth, patient outcomes were limited to health care utilization and mortality. While these clinical endpoints are significant, they do not capture the full spectrum of patient experiences and health status. Fifth, there is limited literature describing the causal mechanisms linking SDoH to patient outcomes in the LTACH setting. Only three of the five SDoH domains were identified in the reviewed studies, which limits the authors’ ability to understand the influence of all SDoH domains on LTACH discharge outcomes. In particular, the impact of community- and institutional-level SDoH on patient outcomes is absent from the findings. These factors represent a gap in the literature that could benefit from further research.

Future research should comprehensively examine how SDoH and CCI influence various patient outcomes, including quality of life and symptom burden. It is equally important to investigate the challenges and adaptive strategies of family members who provide patient support. Using qualitative and quantitative methods will help clarify the impacts of SDoH in this population. Moreover, administrative datasets such as the Long-Term Care Hospital Continuity Assessment Record and Evaluation Data Set (LCDS) [57] can facilitate exploration of how SDoH factors are linked to illness severity, prolonged hospitalization, and mortality.

## Conclusion

Our review identified three SDoH domains and four SDoH variables, with race being the most frequently reported. Given the limited literature on this topic, the review findings were insufficient to describe the complex interplay between SDoH variables and key patient outcomes. Additionally, the degree to which the variables under each SDoH domain have been studied may be insufficient to provide representative information; therefore, caution is strongly recommended when interpreting these findings. SDoH plays a significant role in patient outcomes; thus, understanding and addressing these factors can help to promote health equity and foster optimal health outcomes among patients in the LTACH setting.

## Supporting information

S1 Appendix Search strategies. (DOCX)
